# Supplementary material for: Unveiling the Venom Composition of the Colombian Coral Snakes Micrurus helleri, M. medemi, and M. sangilensis
Source: Toxins (Basel). 2023 Oct 24;15(11):622. doi: 10.3390/toxins15110622 (PMC10674450; doi:10.3390/toxins15110622)
Supplement: Supplementary file 1 [file toxins-15-00622-s001.zip › toxins-2493250-supplementary.pdf]

Article

Unveiling the venom composition of the Colombian coral snakes *Micrurus helleri, M. medemi,* and *M. sangilensis*

Supplementary Table

Supplemental Table 1. Predominant proteomic composition of various coral snake species in the Americas

Species	PLA_2 (%)	3FTx (%)	Location	Dominance	Group	Other toxins		Reference
						SVMP (%)	SVSP (%)	
M. alleni	10.9	77.3	Caribbean Costa Rica	3FTx	Monadal	1.2	ND	[1]
M. altirostris	13.7	79.5	South Brazil	3FTx	Triadal	0.9	ND	[2]
M. clarki	36.5	48.2	Costa Rica Pacific	3FTx	Monadal	1.6	1.0	[3]
M. corallinus	11.9	81.7	Brazil	3FTx	Monadal	2.4	ND	[2]
M. dumerilii	52.0	28.1	Antioquia, Colombia	PLA_2	Monadal	1.8	1.9	[4]
M. frontalis	49.2	42.4	Sao Paulo, Brazil	PLA_2	Triadal	0.96	ND	[5]
M. fulvius	58.3	24.9	Florida, USA	PLA_2	Monadal	NQ	ND	[6]
M. helleri	62.5	21.1	Leticia, Colombia	PLA_2	Triadal	ND	ND	[7]
M. helleri	72.1	17.8	Nangaritza, Ecuador	PLA_2	Triadal	ND	ND	[8]
M. helleri	40.6	14.1	Villagarzón, Putumayo, Colombia	PLA_2	Triadal	ND	ND	This work
M. ibiboboca	66.5	13.5	Bahía, Brazil	PLA_2	Triadal	2.5	ND	[7]
M. lemniscatus carvalhoi	20.2	76.7	Sao Paulo, Brazil	3FTx	Triadal	1.1	ND	[7]
M. lemniscatus carvalhoi	22.1	71.3	Rio de Janeiro, Brazil	3FTx	Triadal	0.8	ND	[7]
M. medemi	43.1	17.7	Villavicencio, Meta, Colombia	PLA_2	Monadal	ND	ND	This work
M. mipartitus	19.1	63.4	Esmeraldas, Ecuador	3FTx	Bicolor	ND	ND	[8]
M. mipartitus	29.0	61.1	Antioquia, Colombia	3FTx	Bicolor	1.6	1.3	[9]
M. mipartitus	8.2	83.0	Costa Rica	3FTx	Bicolor	3.6	ND	[9]
M. mipartitus (M. multifasciatus)	8.2	83.0	Costa Rica	3FTx	Bicolor	3.6	ND	[9]

M. mosquitensis	55.6	22.5	Caribbean Costa Rica	PLA_2	Monadal	2.6	0.5	[1]
M. nigrocinctus	48.0	38.0	Costa Rica	PLA_2	Monadal	4.3	0.7	[10]
M. pyrrhocryptus	17.0	27.0	Cordoba, Argentina	3FTx	Triadal	12.0	7.0	[11]
M. ruatanus	29.8	46.4	Roatan, Honduras (Confiscated)	3FTx	Monadal	2.7	1.2	[12]
M. sangilensis	30.4	17.7	San Gil, Santander, Colombia	PLA_2	Monadal	ND	ND	This work
M. spixii	37.4	56.5	Pará, Brazil	3FTx	Triadal	ND	ND	[5]
M. surinamensis	4.2	95.4	Rondonia, Brazil	3FTx	Triadal	ND	ND	[5]
M. tener	45.6	37.5	Tamaulipas, Mexico	PLA_2	Monadal	ND	ND	[13]
M. tschudii	4.1	95.2	Peruvian Pacific	3FTx	Triadal	ND	ND	[14]
M. yatesi	54.7	20.2	Pacific Costa Rica	PLA_2	Monadal	7.6	1.4	[15]

PLA_2: Phospholipases A_2, 3FTx: three-finger toxins, SVMP: snake venom metalloproteinases, SVSP: snake venom serine proteases, ND: Not determined, NQ: Determined but not quantified.

References

1. Fernández, J.; Vargas-Vargas, N.; Pla, D.; Sasa, M.; Rey-Suárez, P.; Sanz, L.; Gutiérrez, J.M.; Calvete, J.; Lomonte, B. Snake venomics of Micrurus alleni and Micrurus mosquitensis from the Caribbean region of Costa Rica reveals two divergent compositional patterns in New World elapids. *Toxicon* **2015**, *107*, 217–233, doi:10.1016/j.toxicon.2015.08.016.
2. Corrêa-Netto, C.; Junqueira-De-Azevedo, I. de L.M.; Silva, D.A.; Ho, P.L.; Leitão-De-Araújo, M.; Alves, M.L.M.; Sanz, L.; Foguel, D.; Zingali, R.B.; Calvete, J. Snake venomics and venom gland transcriptomic analysis of Brazilian coral snakes, Micrurus altirostris and M. corallinus. *J. Proteomics* **2011**, *74*, 1795–1809, doi:10.1016/j.jprot.2011.04.003.
3. Lomonte, B.; Sasa, M.; Rey-Suárez, P.; Bryan, W.; Gutiérrez, J.M.J.M. Venom of the coral snake Micrurus clarki: Proteomic profile, toxicity, immunological cross-neutralization, and characterization of a three-finger Toxin. *Toxins (Basel).* **2016**, *8*, doi:10.3390/toxins8050138.
4. Rey-Suárez, P.; Núñez, V.; Fernández, J.; Lomonte, B.; Nuñez, V.; Fernández, J.; Lomonte, B. Integrative characterization of the venom of the coral snake Micrurus dumerilii (Elapidae) from Colombia: Proteome, toxicity, and cross-neutralization by antivenom. *J. Proteomics* **2016**, *136*, 262–273, doi:10.1016/j.jprot.2016.02.006.
5. Sanz, L.; de Freitas-Lima, L.N.; Quesada-Bernat, S.; Graça-de-Souza, V.K.; Soares, A.M.; Calderón, L. de A.; Calvete, J.J.; Caldeira, C.A.S. Comparative venomics of Brazilian coral snakes: Micrurus frontalis, Micrurus spixii spixii, and Micrurus surinamensis. *Toxicon* **2019**, *166*, 39–45, doi:10.1016/j.toxicon.2019.05.001.
6. Vergara, I.; Pedraza-Escalona, M.; Paniagua, D.; Restano-Cassulini, R.; Zamudio, F.; Batista, C.V.F.; Possani, L.D.; Alagón, A. Eastern coral snake Micrurus fulvius venom toxicity in mice is mainly determined by neurotoxic phospholipases A2. *J. Proteomics* **2014**, *105*, 295–306, doi:10.1016/j.jprot.2014.02.027.
7. Sanz, L.; Quesada-Bernat, S.; Ramos, T.; Casais-e-Silva, L.L.; Corrêa-Netto, C.; Silva-Haad, J.J.; Sasa, M.; Lomonte, B.; Calvete, J.J. New insights into the phylogeographic distribution of the 3FTx/PLA 2 venom

dichotomy across genus Micrurus in South America. *J. Proteomics* **2019**, *200*, 90–101, doi:10.1016/j.jprot.2019.03.014.
8. Hernández-Altamirano, J.A.; Salazar-Valenzuela, D.; Medina-Villamizar, E.J.; Quirola, D.R.; Patel, K.; Vaiyapuri, S.; Lomonte, B.; Almeida, J.R. First Insights into the Venom Composition of Two Ecuadorian Coral Snakes. *Int. J. Mol. Sci.* **2022**, *23*, doi:10.3390/ijms232314686.
9. Rey-Suárez, P.; Núñez, V.; Gutiérrez, J.M.; Lomonte, B. Proteomic and biological characterization of the venom of the redtail coral snake, Micrurus mipartitus (Elapidae), from Colombia and Costa Rica. *J. Proteomics* **2011**, *75*, 655–667, doi:10.1016/j.jprot.2011.09.003.
10. Fernández, J.; Alape-Girón, A.; Angulo, Y.; Sanz, L.; Gutiérrez, J.M.; Calvete, J.; Lomonte, B.; Fernández, J.; Alape-Girón, A.; Angulo, Y.; et al. Venomic and Antivenomic Analyses of the Central American Coral Snake , Micrurus nigrocinctus (Elapidae). *J. Proteome Res.* **2011**, *10*, 1816–1827, doi:10.1021/pr101091a.
11. Olamendi-Portugal, T.; Batista, C.V.F.; Pedraza-Escalona, M.; Restano-Cassulini, R.; Zamudio, F.Z.; Benard-Valle, M.; Rafael de Roodt, A.; Possani, L.D. New insights into the proteomic characterization of the coral snake Micrurus pyrrhocryptus venom. *Toxicon* **2018**, *153*, 23–31, doi:10.1016/j.toxicon.2018.08.003.
12. Lippa, E.; Török, F.; Gómez, A.; Corrales, G.; Chacón, D.; Sasa, M.; Gutiérrez, J.M.; Lomonte, B.; Fernández, J. First look into the venom of Roatan Island's critically endangered coral snake Micrurus ruatanus: Proteomic characterization, toxicity, immunorecognition and neutralization by an antivenom. *J. Proteomics* **2019**, *198*, 177–185, doi:10.1016/j.jprot.2019.01.007.
13. Bénard-Valle, M.; Carbajal-Saucedo, A.; de Roodt, A.; López-Vera, E.; Alagón, A. Biochemical characterization of the venom of the coral snake Micrurus tener and comparative biological activities in the mouse and a reptile model. *Toxicon* **2014**, *77*, 6–15, doi:10.1016/j.toxicon.2013.10.005.
14. Sanz, L.; Pla, D.; Pérez, A.; Rodríguez, Y.; Zavaleta, A.; Salas, M.; Lomonte, B.; Calvete, J. Venomic analysis of the poorly studied desert coral snake, Micrurus tschudii tschudii, supports the 3FTx/PLA2 dichotomy across Micrurus venoms. *Toxins (Basel).* **2016**, *8*, 9–12, doi:10.3390/toxins8060178.
15. Mena, G.; Chaves-Araya, S.; Chacón, J.; Török, E.; Török, F.; Bonilla, F.; Sasa, M.; Gutiérrez, J.M.; Lomonte, B.; Fernández, J. Proteomic and toxicological analysis of the venom of Micrurus yatesi and its neutralization by an antivenom. *Toxicon X* **2022**, *13*, doi:10.1016/j.toxcx.2022.100097.
